# Residual Activatability of Circulating Tfh17 Predicts Humoral Response to Thymodependent Antigens in Patients on Therapeutic Immunosuppression

**DOI:** 10.3389/fimmu.2018.03178

**Published:** 2019-02-05

**Authors:** Suzan Dahdal, Carole Saison, Martine Valette, Emmanuel Bachy, Nicolas Pallet, Bruno Lina, Alice Koenig, Guillaume Monneret, Thierry Defrance, Emmanuel Morelon, Olivier Thaunat

**Affiliations:** ^1^French National Institute of Health and Medical Research (Inserm) Unit 1111, Lyon, France; ^2^Department of Transplantation, Nephrology and Clinical Immunology, Hospices Civils de Lyon, Edouard Herriot University Hospital, Lyon, France; ^3^Hospices Civils de Lyon, Croix-Rousse University Hospital, Infectious Agents Institute (IAI) Laboratory of Virology-National Reference Center for Respiratory Viruses (Including Influenza), Lyon, France; ^4^Department of Hematology, Hospices Civils de Lyon, Lyon Sud University Hospital, Pierre Bénite, France; ^5^Claude Bernard University (Lyon 1), Lyon, France; ^6^Laboratory of Biochemistry, Assistance Publique-Hôpitaux de Paris, Georges Pompidou Hospital, Paris, France; ^7^Paris Descartes University, Paris, France; ^8^Laboratory of Immunology, Hospices Civils de Lyon, Edouard Herriot University Hospital, Lyon, France

**Keywords:** transplantation, donor-specific antibodies, biomarkers, Tfh, immunosuppression

## Abstract

The generation of antibodies against protein antigens (such as donor-specific HLA molecules) requires that T follicular helper cells (Tfh) provide help to B cells. Immunosuppressive (IS) armamentarium prevents T cell activation, yet a significant proportion of renal transplant patients develop donor-specific antibodies (DSA), which suggests that IS drugs do not efficiently block T follicular helper cells. To test this hypothesis, the number of circulating Tfh, their polarization profile, and ability to up-regulate (i) the co-stimulatory molecules CD40L and ICOS, and (ii) the activation marker CD25, following *in vitro* stimulation in presence of IS drugs, were compared between 36 renal transplant patients (6–72 months post transplantation) and nine healthy controls. IS drugs reduced the number of Tfh1 and 2 but had little impact on Tfh17, which was the dominant subset in transplant patients. Although, IS drugs decreased activation-induced expression of co-stimulatory molecules by Tfh, the impact was highly variable between individuals. Furthermore, 20% of transplant patients displayed normal expression of CD25 on Tfh following *in vitro* stimulation (i.e., “residual activatability”). To test whether residual activatability of Tfh correlates with antibody response against thymo-dependent antigens we took advantage of the 2015 influenza vaccination campaign, which provided a normalized setting for antigenic stimulation. In line with our hypothesis, responders to influenza vaccine exhibited significantly higher percentage of CD25-expressing Tfh17 after *in vitro* stimulation. A results that was confirmed retrospectively in nine transplanted patients at the time of first DSA detection. We concluded that “residual activatability” of Tfh17 might be used as a non-invasive biomarker to identify transplant patients at higher risk to develop DSA under immunosuppression. If validated in larger studies, this assay might help optimizing the prevention of DSA through personalized adaptation of immunosuppressive regimen.

## Introduction

Solid organ transplantation is the best (often the only) therapeutic option to restore the physiologic functions of a defective vital organ. Although transplantation saves thousands of lives and transforms the quality of life of thousands more, long-term success remains limited by the progressive decline of graft function. Indeed, advances over the last decades have had almost negligible impact on the rate of late graft loss (1). Expending the duration of graft function therefore currently represents a major challenge in the field of transplantation (2).

The pathophysiology of the progressive and irreversible loss of graft function is highly complex, but accumulating evidence points at the crucial role of antibodies directed against donor-specific alloantigens (donor-specific antibodies: DSA) (3, 4). Several clinical studies have unraveled a strong epidemiological link between appearance of DSA in the circulation and subsequent transplant failure (5–8). The causal role of DSA has then been formerly established by experimental studies demonstrating that repeated passive administrations of DSA were sufficient to trigger the development of histological lesions in the vasculature of allogenic cardiac grafts transplanted to immunodeficient mice (9).

The current consensus for antibody-mediated rejection (AMR) treatment associates rapid depletion of circulating DSA with plasmapheresis and a combination of corticosteroid and high dose intravenous immunoglobulins (10, 11). Because this costly and tedious therapeutic approach has no direct impact on DSA-producing plasma cells, it only has (at best) a suspensive effect on antibody-mediated graft destruction. As a result, reported 3 years graft survival for AMR is currently estimated below 50% (12, 13).

In the absence of efficient curative treatment for AMR, primary prevention of DSA generation by therapeutic immunosuppression remains the best prospect to improve long-term outcome of solid organ transplantation. Yet, modern immunosuppression regimen is not fully effective to block humoral alloimmune response (14, 15), as shown by the prevalence of *de novo* DSA, which is estimated 10–25% 5 years post-transplantation (16, 17).

Highly polymorphic HLA proteins, which represent the most documented targets of DSA, are prototypic T-cell-dependent antigens. It implies that donor-HLA specific B cells are critically dependent upon the help of CD4+ T cells to differentiate into DSA-producing plasma cells (18). In support with this dogma, we have recently obtained experimental data demonstrating the total abrogation of DSA responses (both naive and memory) in the absence of CD4+ T cells (19). Basic immunological studies have identified the subset of CD4+ T cells (named T follicular helper), specialized for providing help to B cells in secondary lymphoid organs during antibody-responses (20, 21). The fact that some transplanted patients develop DSA under therapeutic immunosuppression suggests that immunosuppressive drugs (either because of poor adherence or insufficient dosing) insufficiently block helper function of Tfh in these patients (19, 22, 23). Interestingly, a recent work has shown that human blood CXCR5+ CD4+ T cells are the circulating equivalents of Tfh (24), offering a window of opportunity to monitor this cell subset in patients.

In this translational study, aiming at gaining insights on the impact of therapeutic immunosuppression on Tfh, we compared the characteristics of circulating Tfh (cTfh) of renal recipients at different time post-transplantation with cTfh of healthy volunteers. We then tested whether this non-invasive monitoring could predict antibody response to a model thymodependent antigen: influenza hemaglutinin.

## Materials and Methods

### Study Population

The study was approved by the “Comité de Protection des Personnes Sud-Est IV” (ref#L15-166) and all patients signed a consent form to participate in this study.

A total of 36 renal transplant patients and 9 healthy volunteers were prospectively recruited.

The inclusion and exclusion criteria were as follows. Inclusion criteria: age 18–70 years, patient that had received a first isolated renal transplant or a first combined kidney-pancreas transplantation between 6 and 72 months before, no circulating anti-HLA antibodies, signed informed consent form. Exclusion criteria: second (or more) transplantation, fever or symptoms of flu or other infectious disease, hypersensitivity to any components of influvac® vaccine, patients who have received blood products or intravenous immunoglobulins in the past 3 months, pregnancy, ongoing rejection.

The nature of the immunosuppressive drugs and their trough levels were recorded at inclusion, as well as relevant clinical data, such as proteinuria and estimated glomerular filtration rate (eGFR), estimated by the CKD-EPI formula.

### Cell Culture

Blood was collected immediately prior influenza vaccination and between 21 and 28 days later. Peripheral blood mononuclear cells (PBMC) and plasma were isolated by Ficoll gradient centrifugation. PBMCs were cultured 1 h at 37°C in petri dishes and adherent cells were discarded. One million of non-adherent cells were cultured 24 h at 37°C in 5% CO_2_ in 1 mL of their own plasma with and without the anti-CD3/CD28 beads (Gibco Dynabeads®). Importantly, this assay was run in the patients' own plasma, i.e., in presence of clinically relevant concentration of immunosuppressive drugs.

### Flow Cytometry

After 24 h of culture, the anti-CD3/CD28 beads were removed using a magnet and the cells were stained 30 min at room temperature in the dark with fluorescent antibodies according to the manufacturer's instructions. The following antibodies were used: antibodies against CD3 (UCHT1), CD4 (SK3), CXCR5 (RF8B2), CXCR3 (1C3), CCR6 (11A9), CD25 (2A3), CD40L (TRAP1), ICOS (ISA-3), (All from BD Biosciences) and the viability dye LIVE/DEAD Aqua (Invitrogen). Then cells were fixed with Cytofix/Cytoperm® fixation/ permeabilization kit (BD Biosciences).

A FACS ARIA II flow cytometer was used for flow cytometry. Data were analyzed with BD FACS Diva (BD Biosciences) and FlowJo® software (FlowJo, LLC). To ensure the comparability of data, all samples were run on the same instrument, which was calibrated with Rainbow Calibration Particles® before each acquisition (SpheroTech).

The absolute number of cTfh cells was calculated by using the lymphocyte count of the hemogram test performed the day of the blood sampling and flow cytometry data obtained in the non-activated lymphocytes condition.

### Hemagglutination Inhibition Assay

The hemagglutinin surface protein of the influenza virus has the property to bind to sialic acid receptors on erythrocytes, causing the formation of a lattice. This property is called hemagglutination, and is the basis of the Hemagglutination-Inhibition (HI) assay.

The HI assay involves the interaction of influenza virus to anti-hemagglutinin antibodies or to the red blood cells. A fixed amount of each vaccine strain was added to serial dilution of the serum and the plate was incubated for 1 h. Finally, red blood cells were added and the plate was incubated for one additional hour. The red blood cells that are not bound by influenza virus sink to the bottom of a well and form a button. The red blood cells that are attached to virus particles form a lattice that coats the well (hemagglutination). When antibodies against influenza virus are present, they prevent the attachment of the virus to red blood cells and therefore inhibit the hemagglutination. The highest dilution of serum that prevents hemagglutination defines the HI titer of the serum and corresponds to the antibody titer against the tested influenza virus strain.

### Statistical Analyses

Categorical variables were expressed as percentages and Continuous variables were expressed as mean ± standard deviation (SD). Differences between the groups were evaluated by: Mann-Whitney test, fisher's exact test, unpaired *t*-test, or one-way ANOVA followed by a Dunn's multiple comparisons test, according to the size of the groups and the distribution of the variable.

All the tests used were two-sided. The test used for comparison is indicated in the figure legends.

The differences between the groups were considered statistically significant for *p* < 0.05 and were reported with asterisk symbols (^*^p < 0.05; ^**^p < 0.01; ^***^p < 0.001; ^****^p < 0.0001).

## Results

### Population of the Study

A total of 36 renal transplant patients followed in Lyon University Hospital were prospectively recruited in this study.

Although there is no reliable tool to measure the depth of therapeutic immunosuppression, it is widely accepted that immunosuppression is maximal during the 12 months after transplantation, the period when drug trough levels are set at the highest (25). This is particularly true for patients that receive a depleting agent as induction [for whom immune reconstitution can take up to 1 year (26)]. To take this heterogeneity into account, the cohort of transplant patients was split in two groups according to the time elapsed since transplantation: early Tx (6–12 months) and late Tx (>12–72 months). The characteristics of the two groups are presented in Table 1.

**Table 1:** Baseline characteristics of the study population.

	**Early transplanted *n* = 18**	**Late transplanted *n* = 18**	*p*-value^*^
**CHARACTERISTICS AT TIME OF TRANSPLANTATION**			
**Recipient**			
Age at transplantation (years)	54 ± 14	50 ± 14	0.47
Origin of renal disease, *n* (%)			0.61
Glomerulonephritis	1 (6)	3 (17)
Diabetes mellitus	1 (6)	4 (22)
Vascular	8 (44)	3 (17)
Genetic	4 (22)	3 (17)
Uropathy	1 (6)	1 (6)
Undetermined	3 (17)	4 (22)
Duration of dialysis (months)	20 ± 18	31 ± 41	0.79
**DONOR**			
Donor age (years)	53 ± 14	46 ± 16	0.18
Donor sex, men, *n* (%)	12 (67)	14 (78)	0.71
Deceased donor, *n* (%)	15 (83)	17 (94)	0.60
Extended criteria donor, *n* (%)	7 (39)	6 (33)	1.0
**TRANSPLANTATION**			
Number of HLA-A, B, C, DR, DQ mismatches	5.5 ± 2.1	5.8 ± 1.6	0.59
Combined kidney-pancreas transplantation, n (%)	0 (0)	3 (17)	0.22
Cold ischemia time (min)	726 ± 479	768 ± 427	0.90
Delayed graft function, *n* (%)	2 (11)	2 (11)	1.0
**IMMUNOSUPPRESSION**			
Induction therapy			
Thymoglobuline, *n* (%)	12 (67)	15 (83)	0.44
Maintenance regimen			
Tacrolimus, *n* (%)	13 (72)	17 (94)	0.17
Ciclosporin, *n* (%)	5 (28)	1 (6)	0.17
mTOR-Inhibitors, *n* (%)	3 (17)	1 (6)	0.60
Mycophenolate, *n* (%)	17 (94)	16 (89)	1.0
Azathioprin, *n* (%)	0 (0)	1 (6)	1.0
Prednisone, *n* (%)	11 (61)	14 (78)	0.47
**CHARACTERISTICS AT TIME OF VACCINATION**			
Time elapsed since transplantation (months)	10 ± 4	37 ± 14	< 0.0001
eGFR (ml/min/1.73m^2^)	56 ± 21	65 ± 23	0.22
Proteinuria >0.5 g/24 h, *n* (%)	3 (17)	4 (22)	0.91

* *Unpaired t-test was used for comparison of continuous variables and Fisher's exact test was used for comparison of proportions*.

*mTOR, mammalian target of rapamycin; eGFR, estimated glomerular filtration rate*.

Renal recipients were compared to nine healthy controls (Ctl). Comparing them to patients on the waiting list would indeed not have allowed disentangling the impact of immunosuppressive drugs from that of the correction of end stage renal failure, which has also a major impact on the immune system (27).

### Impact of Therapeutic Immunosuppression on cTfh Profile

A recent study has reported that circulating Tfh (cTfh), defined as CD4+CD3+ T cells expressing CXCR5 (the chemokine receptor required to respond to CXCL13, the homeostatic chemokine of B cell zone of secondary lymphoid organs), can be detected in the circulation of autoimmune patients (24). Other works have shown that cTfh could be distributed into three subpopulations with distinct polarization profiles, according to the expression of two chemokine receptors CCR6 and CXCR3: Tfh1 (CCR6- CXCR3+), Tfh2 (CCR6- CXCR3-), and Tfh17 (CCR6+) (28).

**Figure 1:**
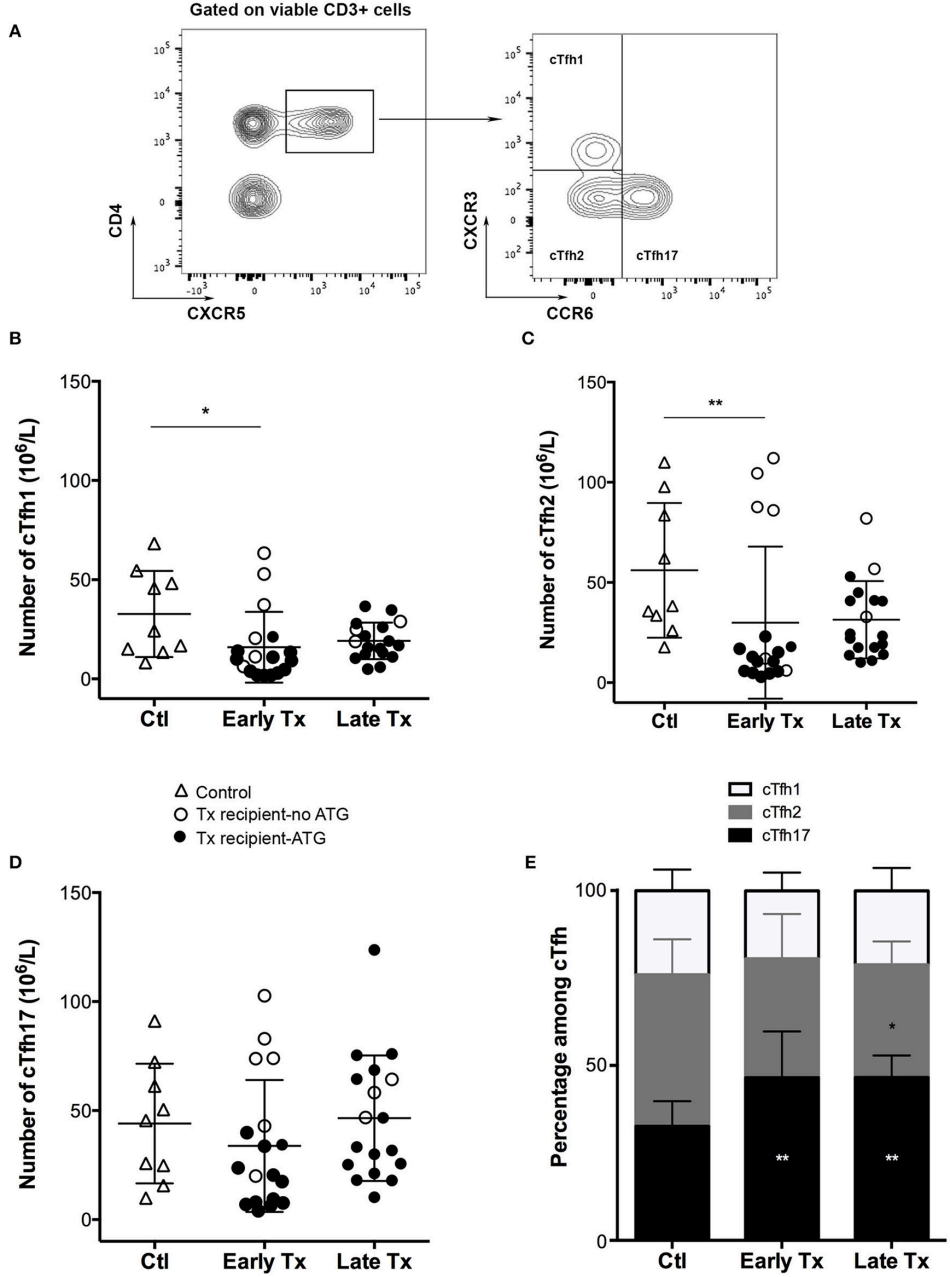
Comparison of the static profile of cTfh of controls and transplants patients. **(A)** PBMC of patients were collected just before vaccination. Flow cytometry gating strategy used to identify the three subsets of circulating T follicular helper (cTfh). **(B–D)** The number cTfh of each of the 3 subsets **(B**: cTfh1; **C**: Tfh2; **D**: cTfh17) was enumerated among the PBMCs of healthy volunteers (*n* = 9; controls, Ctl; open triangles), and transplant patients (*n* = 36, circles). The nature of induction immunosuppressive therapy received by transplant patient is indicated. Transplant patients were distributed into two groups according to the time elapsed since transplantation: (i) 6–12 months: early post-transplantation (Early Tx; *n* = 18), and (ii) >12–72 months: late post-transplantation (Late Tx; *n* = 18). Each symbol represents a patient, mean ± SD is indicated. ^*^p < 0.05; ^**^p < 0.01; ANOVA, Dunn's multiple comparisons test. **(E)** The proportion (mean ± SD) of each of the three subsets of cTfh is compared between the three groups. ^*^p < 0.05; ^**^p < 0.01; Mann-Whitney test.

Using the same flow cytometry strategy (Figure 1A), we detected the presence of the three cTfh subsets among the PBMC of both controls and transplant patients (Figures 1B–D). The absolute number of cTfh was highly variable between individuals, including in the control group. The number of cThf1 and cTfh2 was significantly decreased in the group early Tx as compared with controls but this was likely due to the high proportion of patients that received a depleting induction with thymoglobulin (Figures 1B,C). Interestingly immunosuppression did not have the same impact on cTfh17, the number of which remained similar to that observed in healthy controls (Figure 1D). As result, cTfh17 was the dominant subset in early and late Tx groups, accounting for ~50% of circulating Tfh (Figure 1E). Importantly, Tfh17 is recognized as the most effective subsets to provide the help required for B cells differentiation into plasma cells (28).

### Impact of Therapeutic Immunosuppression on cTfh Functionality

**Figure 2:**
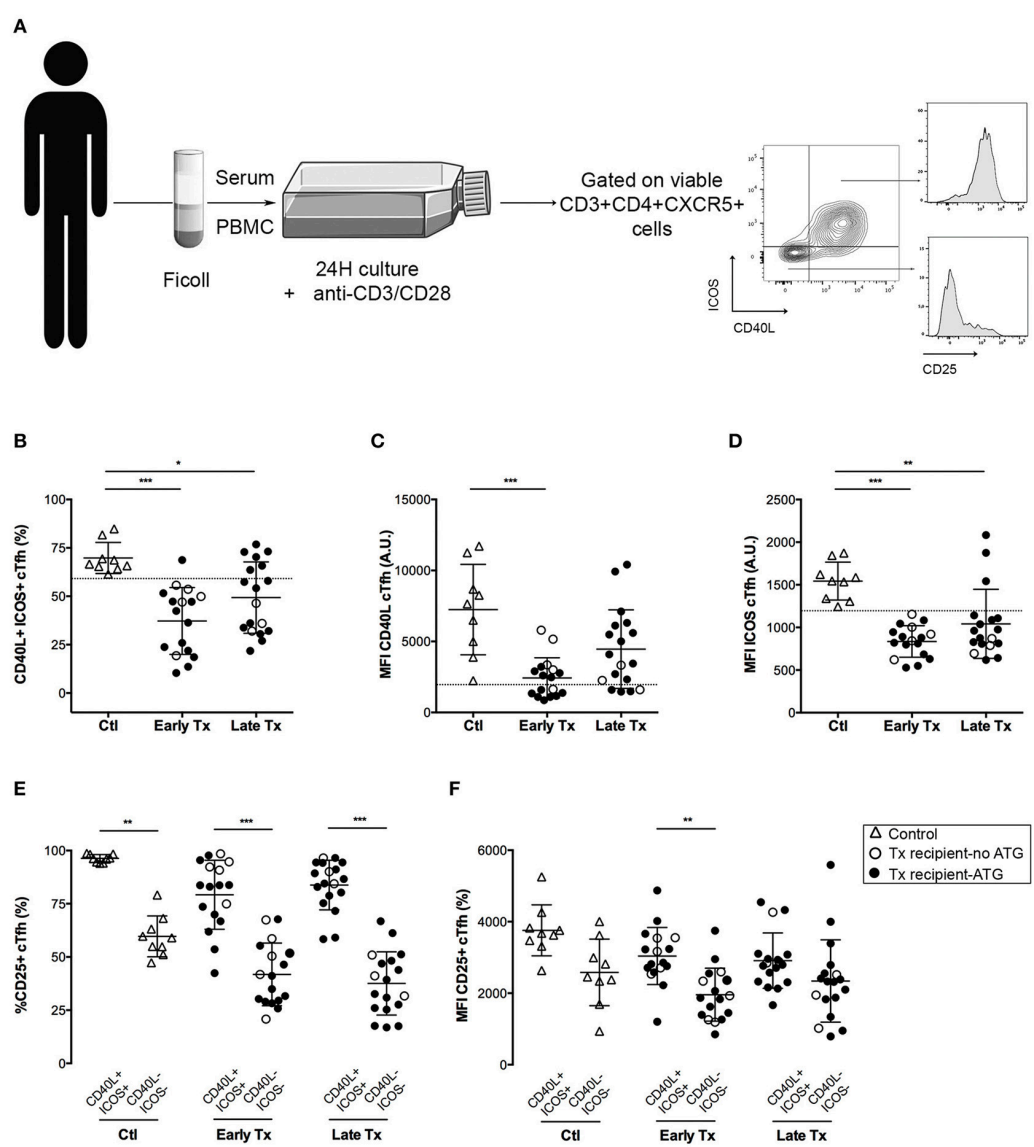
Comparison of the functional profile of cTfh of controls and transplants patients. **(A)** Graphical summary of the procedure used to evaluate the functionality of cTfh. PBMC of patients were collected just before vaccination. The level of expression of CD40L ICOS and CD25 was measured by flow cytometry on the surface of cTfh, after 24 h stimulation with anti-CD3/CD28 microbeads in patient's own serum. **(B)** The percentage of CD40L^+^ICOS^+^ cTfh was enumerated by flow cytometry after 24 h of *in vitro* stimulation in healthy volunteers (*n* = 9; controls, Ctl; open triangles), and transplant patients (*n* = 36, circles). The nature of induction immunosuppressive therapy received by transplant patient is indicated. Transplant patients were distributed into two groups according to the time elapsed since transplantation: (i) 6–12 months: early post-transplantation (Early Tx; *n* = 18), and (ii) >12–72 months: late post-transplantation (Late Tx; *n* = 18). Dotted line indicates the lower value observed in control patients. **(C,D)** The level of expression of CD40L **(C)** and ICOS **(D)**, as reflected by mean fluorescence intensity (MFI), was measured by flow cytometry after 24 h of *in vitro* stimulation of cTfh and compared between the 3 groups. **(E)** The percentage of CD25-positive cells was compared between CD40L^+^ICOS^+^ and CD40L^−^ICOS^−^ cTfh subsets in the 3 groups. **(F)** The level of expression of CD25 was compared between CD40L^+^ICOS^+^ and CD40L^−^ICOS^−^ cTfh subsets in the three groups. Each symbol represents a patient, mean ± SD is indicated. ^*^p < 0.05; ^**^p < 0.01; ^***^p < 0.001; ANOVA, Dunn's multiple comparisons test.

To test the impact of therapeutic immunosuppression on cTfh, PBMCs of patients were cultured with anti-CD3/CD28 microbeads in the presence of patient's own plasma (containing relevant concentration of IS drugs, Figure 2A).

Tfh provide help to B cells through a variety of surface molecules, including CD40L (29), and ICOS (30). *In vitro* activation of cTfh promoted the expression of both CD40L and ICOS (Figure 2A). Triple maintenance immunosuppression significantly decreased the proportion of CD40L^+^ICOS^+^ cTfh (Figure 2B), as well as the level of expression of these two co-stimulatory molecules (Figures 2C,D). As expected, the impact of therapeutic immunosuppression was more pronounced for the group early Tx, in which the maintenance regimen is the stronger. More interesting was the heterogeneity of individual profiles: some transplanted patients exhibiting a CD40L and ICOS up-regulation almost comparable to controls (Ctl) (Figures 2B–D).

CD40L and ICOS are not the only molecules through which Tfh help B cells to differentiate into antibody-producing plasma cells (30).

Because monitoring each helper pathways for each cTfh subset in each transplanted patient would not be a realistic approach to develop a biomarker in the clinic, we sought for a simple unique molecular marker that could be easily detected on cell surface and could be used as surrogate for cTfh functionality. We observed that the activation marker CD25 (the α chain of IL-2 receptor) was almost constantly expressed by functional (i.e., CD40L^+^ICOS^+^) cTfh but not by the cTfh that did not up-regulate the expression of co-stimulatory molecules after *in vitro* stimulation (Figures 2A,E). Although, the same trend was observed for the level of expression of CD25 (as assessed by the MFI), this variable was less reliable to discriminate functional vs. non-functional cTfh (Figure 2F).

### Impact of Therapeutic Immunosuppression on cTfh Activatability

**Figure 3:**
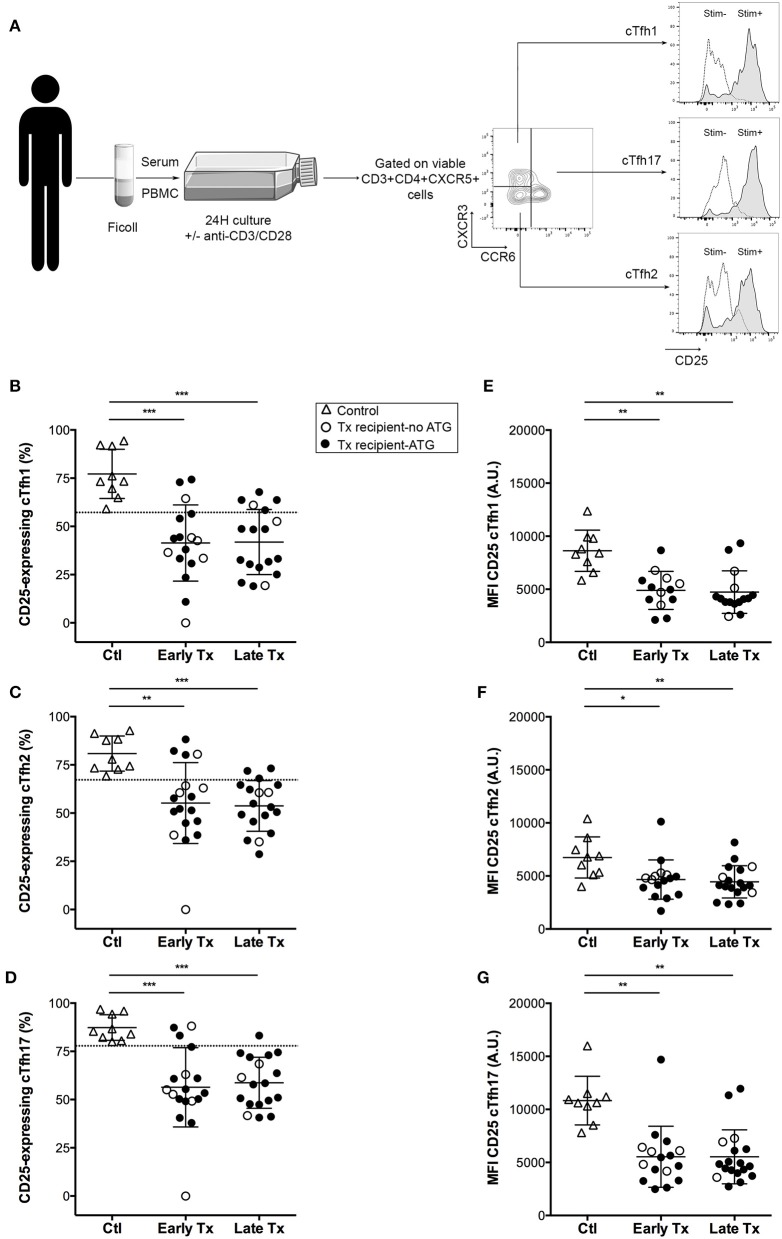
Comparison of the activation profile of cTfh of controls and transplants patients. **(A)** Graphical summary of the procedure used to evaluate the residual activatability of the three subsets of cTfh. PBMC of patients were collected just before vaccination. The level of expression of CD25 was measured by flow cytometry on the surface cTfh, after 24 h culture in patient's own serum with (gray profiles, Stim+) or without (open profiles, Stim–) stimulation with anti-CD3/CD28 microbeads. **(B–D)** The percentage of CD25-expressing cTfh of each of the three subsets (**B**: cTfh1; **C**: Tfh2; **D**: cTfh17) was enumerated by flow cytometry after 24 h of *in vitro* stimulation in healthy volunteers (*n* = 9; controls, Ctl; open triangles), and transplant patients (*n* = 36, circles). The nature of induction immunosuppressive therapy received by transplant patient is indicated. Transplant patients were distributed into two groups according to the time elapsed since transplantation: (i) 6–12 months: early post-transplantation (Early Tx; *n* = 18), and (ii) >12–72 months: late post-transplantation (Late Tx; *n* = 18). Dotted line indicates the lower threshold of the percentage of cTfh expressing CD25 after stimulation in control patients. **(E–G)** The level of expression of CD25 (as reflected by mean fluorescence intensity, MFI) was measured by flow cytometry after 24 h of *in vitro* stimulation for each of the three subsets of cTfh (**E**: cTfh1; **F**: Tfh2; **G**: cTfh17) and compared between the three groups. Each symbol represents a patient, mean ± SD is indicated. ^*^p < 0.05; ^**^p < 0.01; ^***^p < 0.001; ^****^p < 0.0001; ANOVA, Dunn's multiple comparisons test.

**Figure 4:**
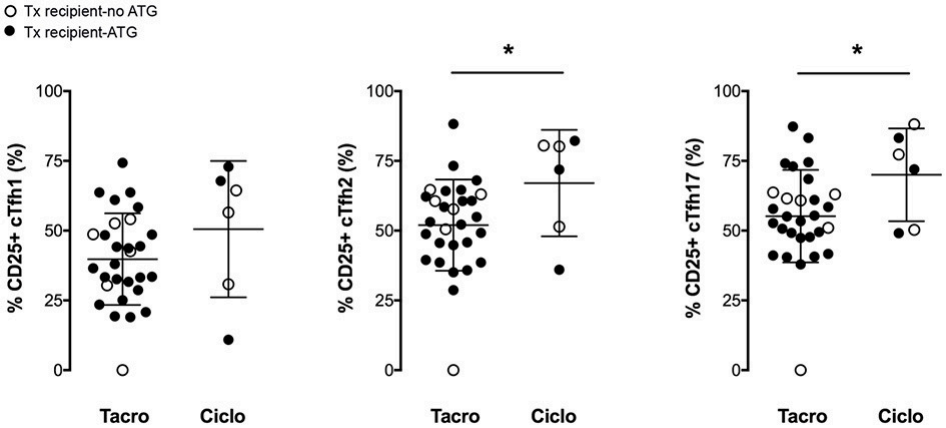
Tacrolimus blocks more efficiently the activation of cTfh. The percentage of CD25-expressing cTfh of each of the three subsets (cTfh1; Tfh2; and cTfh17) was enumerated by flow cytometry after 24 h of *in vitro* stimulation in transplant patients (*n* = 36, circles). The nature of induction immunosuppressive therapy received by transplant patient is indicated. Transplant patients were distributed into two groups according to the CNI used for maintenance immunosuppression: (i) tacrolimus (Tacro; *n* = 30), and (ii) ciclosporin (Ciclo; *n* = 6). Each symbol represents a patient, mean±SD is indicated. ^*^p < 0.05; Unpaired *t*-test.

*In vitro* stimulation of cTfh cells from healthy controls promoted the expression of the activation marker CD25 on all the three subsets (Figures 3A–D). Therapeutic immunosuppression significantly reduced both the percentage of cTfh expressing CD25 (Figures 3B–D) and the level of expression of CD25 (Figures 3E–G) in the two groups (early and late) of transplant patients. Tacrolimus appeared to block more efficiently activation-induced upregulation of CD25 on cTfh (in particular for cTfh2 and cTfh17 subsets) than ciclosporin (Figure 4). The impact of therapeutic immunosuppression was however heterogeneous among patients, a significant proportion of whom, showed “residual activatability” of cTfh. This was illustrated by the fact that, after stimulation, ~20% of transplanted patients had a percentage of CD25+ cTfh similar to what observed in the controls (Figures 3B–D).

Given the importance of Tfh help to generate alloantibodies (19), we hypothesized that higher “residual activatability” of cTfh in transplant patients could correlate with higher risk for *de novo* DSA generation on therapeutic immunosuppression.

### Strategy to Evaluate the Impact of Therapeutic Immunosuppression on Thymo-Dependent Antibody Responses

Evaluating a predictive assay for *de novo* DSA generation is a difficult task. First, appearance of *de novo* DSA is a relatively rare event, the incidence of which is estimated 2–10% per year (16, 17). Second, numerous donor and recipient factors influenced DSA generation (15), which leads to important variability among pairs. This made impossible the study of the direct correlation between parameters of the cTfh profile and the generation of DSA in our cohort. Instead, we took advantage of the 2015 influenza vaccination campaign, which is recommended by international guidelines in transplant patients (31) and offers a “standardized immune stimulation”: i.e., (i) every patient receives the same antigenic challenge, (ii) the timing of immunization is known.

Influenza vaccine formulation includes hemagglutinin proteins, which are known thymo-dependent (TD) antigens (32), from 3 distinct virus strains: in 2015, A/California (H1N1pdm09), A/Switzerland (H3N2), and B/Phuket.

**Figure 5:**
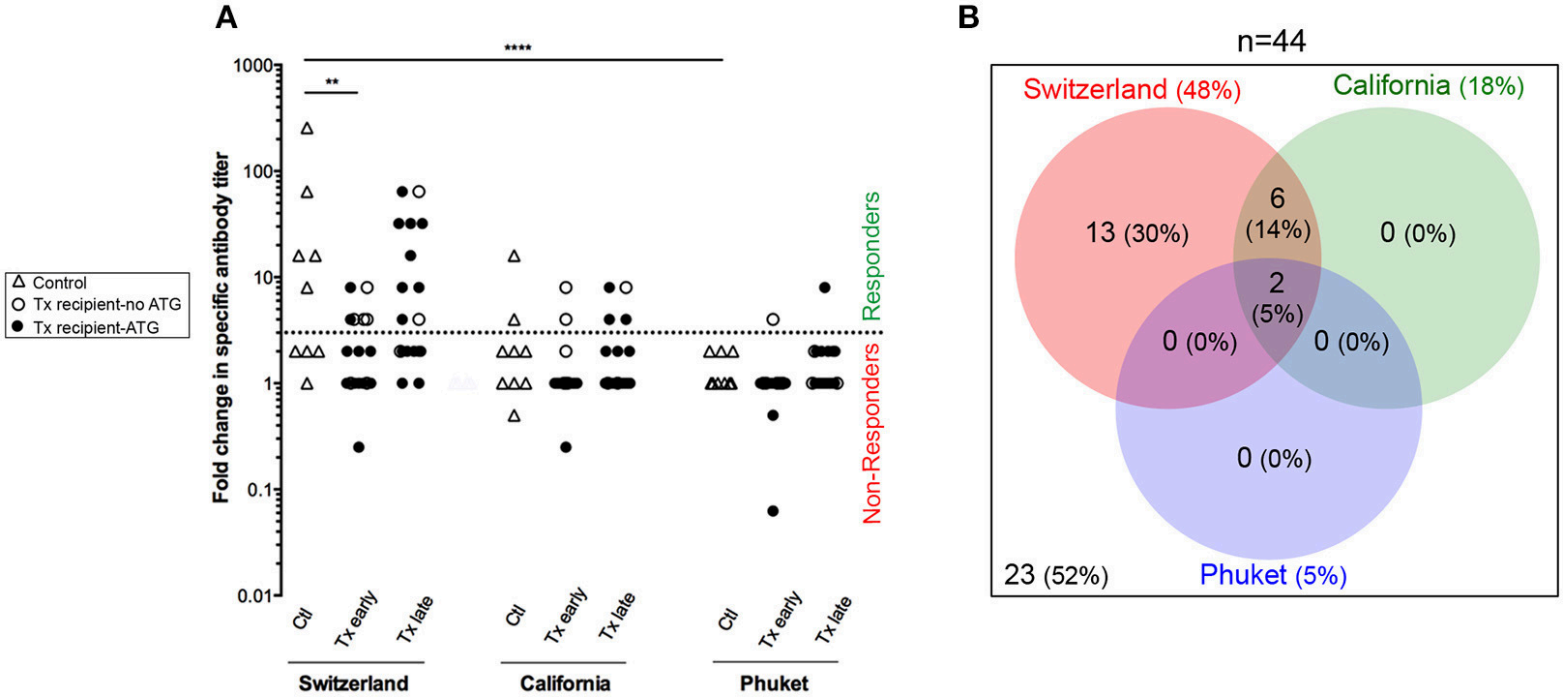
Antibody response to flu vaccination. **(A)** Hemagglutination-Inhibition assay was used to measure the titer of antibodies specific of each strains of influenza virus (Switzerland, California, and Phuket) present in the vaccine. The titer was measured just before and 21–28 days post-vaccination and results are expressed as a ratio of these two values for healthy volunteers (*n* = 9; controls, Ctl; open triangles), and transplant patients (*n* = 36, circles). The nature of induction immunosuppressive therapy received by transplant patient is indicated. Transplant patients were distributed into two groups according to the time elapsed since transplantation: (i) 6–12 months: early post-transplantation (Early Tx; *n* = 18), and (ii) >12–72 months: late post-transplantation (Late Tx; *n* = 18). Dotted line indicates the threshold of fold change in specific antibody titer above which the patient was considered as responder (≥4). **(B)** The Venn diagram summarizes the relations between the responses to the various virus strains.

Anti-hemagglutinin antibody titers were evaluated blindly for each vaccine strain before and at the peak of the response (21–28 days post-vaccination), using the gold standard technique of inhibition of hemagglutination (HI) (33) (Figure 5A). Among the 45 persons enrolled in the study, only one transplanted patient could not be analyzed because his post-vaccination serum couldn't be collected in time. Patients were defined as responder for one strain if the antibody titer against hemagglutinin (HI titer) raised ≥4-folds between pre- and post-vaccination assessment (i.e., patients above the dotted line in Figure 5A).

Antibody responses against the Switzerland and California virus strains were significantly more frequent than response against Phuket virus strain (Figures 5A,B; Chi square test, *p* < 0.0001 and *p* = 0.04, respectively). This is in agreement with the fact that influenza A virus strains (Switzerland and California) are of greater immunogenicity than influenza B virus strains (Phuket) (34). As expected, patients from the group “early Tx” (whom are more immunosuppressed) developed significantly less antibodies to Switzerland hemagglutinin antigen (Figure 5A; ANOVA Tukey's multiple comparisons test, *p* < 0.01**)**.

California virus strain was included in the influenza vaccine since 2010. Because our primary interest was to identify transplant patients at risk for *de novo* DSA generation (i.e., a primary humoral response), we focus the analysis on the Switzerland virus strain, which was selected for the northern hemisphere influenza vaccine formulation for the first time in 2015.

Among kidney transplant recipients (*n* = 35, i.e., excluding the nine control patients and the transplanted patient for whom serum couldn't be collected after vaccination), responders (*n* = 15) and non-responders (*n* = 20) to Switzerland hemagglutinin antigen could not be discriminated on the basis of the clinical characteristics (Table 2) previously associated with humoral response post-transplantation in the literature (15). Mean age and time elapsed since transplantation did not differ between the group of responders and non-responders. Induction therapy was similar between the two groups but (in line with what observed in Figure 4) there was a strong trend for a higher proportion of patients on ciclosporin in the responder group (Table 2).

**Table 2:** Baseline characteristics of responders and non-responders to Switzerland hemagglutinin antigen.

	**NoR** ***n* = 20**	**R** ***n = 15***	*p*-value^*^
**CHARACTERISTICS AT TIME OF TRANSPLANTATION**			
**Recipient**			
Age at transplantation (years)	53 ± 13	51 ± 16	0.74
Origin of renal disease, *n* (%)			0.80
Glomerulonephritis	1 (5)	3 (20)
Diabetes mellitus	3 (15)	2 (13)
Vascular	6 (30)	4 (27)
Genetic	4 (20)	3 (20)
Uropathy	1 (5)	1 (7)
Undetermined	5 (25)	2 (13)
Duration of dialysis (months)	21 ± 20	31 ± 43	0.83
**DONOR**			
Donor age (years)	50 ± 14	48 ± 17	0.71
Donor sex, men, *n* (%)	14 (70)	11 (73)	1.0
Deceased donor, *n* (%)	17 (85)	14 (93)	0.69
Extended criteria donor, n (%)	6 (30)	7 (47)	0.48
**TRANSPLANTATION**			
Number of HLA-A, B, C, DR, DQ mismatches	6.1 ± 1.5	5.2 ± 1.9	0.17
Combined kidney-pancreas transplantation, n (%)	2 (10)	1 (7)	1.00
Cold ischemia time (min)	677 ± 414	806 ± 490	0.40
Delayed graft function, *n* (%)	0 (0)	3 (21)	0.06
**CHARACTERISTICS AT TIME OF VACCINATION**			
Time elapsed since transplantation (months)	23 ± 19	24 ± 15	0.77
**IMMUNOSUPPRESSION**			
**Induction therapy**			
Thymoglobuline, *n* (%)	17 (85)	9 (60)	0.12
**Maintenance regimen**			
Tacrolimus, *n* (%)	19 (95)	10 (67)	0.06
Ciclosporin, *n* (%)	1 (5)	5 (33)
mTOR-Inhibitors, *n* (%)	2 (10)	1 (7)	1.00
Mycophenolate, *n* (%)	18 (90)	14 (93)	1.00
Azathioprin, *n* (%)	1 (5)	0
Prednisone, *n* (%)	12 (60)	12 (80)	0.28
**GRAFT FUNCTION**			
eGFR (ml/min/m^2^)	59 ± 22	61 ± 23	0.78
Proteinuria^**^, *n* (%)	3 (15)	3 (20)	1.00

* Unpaired t-test was used for comparison of continuous variables and Fisher's exact test was used for comparison of proportions.

** Proteinuria is defined as >0.5 g/24 h or >40 mg/mmol creatinine in spot-urine.

*mTOR, mammalian target of rapamycin; eGFR, estimated glomerular filtration rate*.

### Can cTFh Activatability Predict Antibody Response to Thymodependent Antigen?

**Figure 6:**
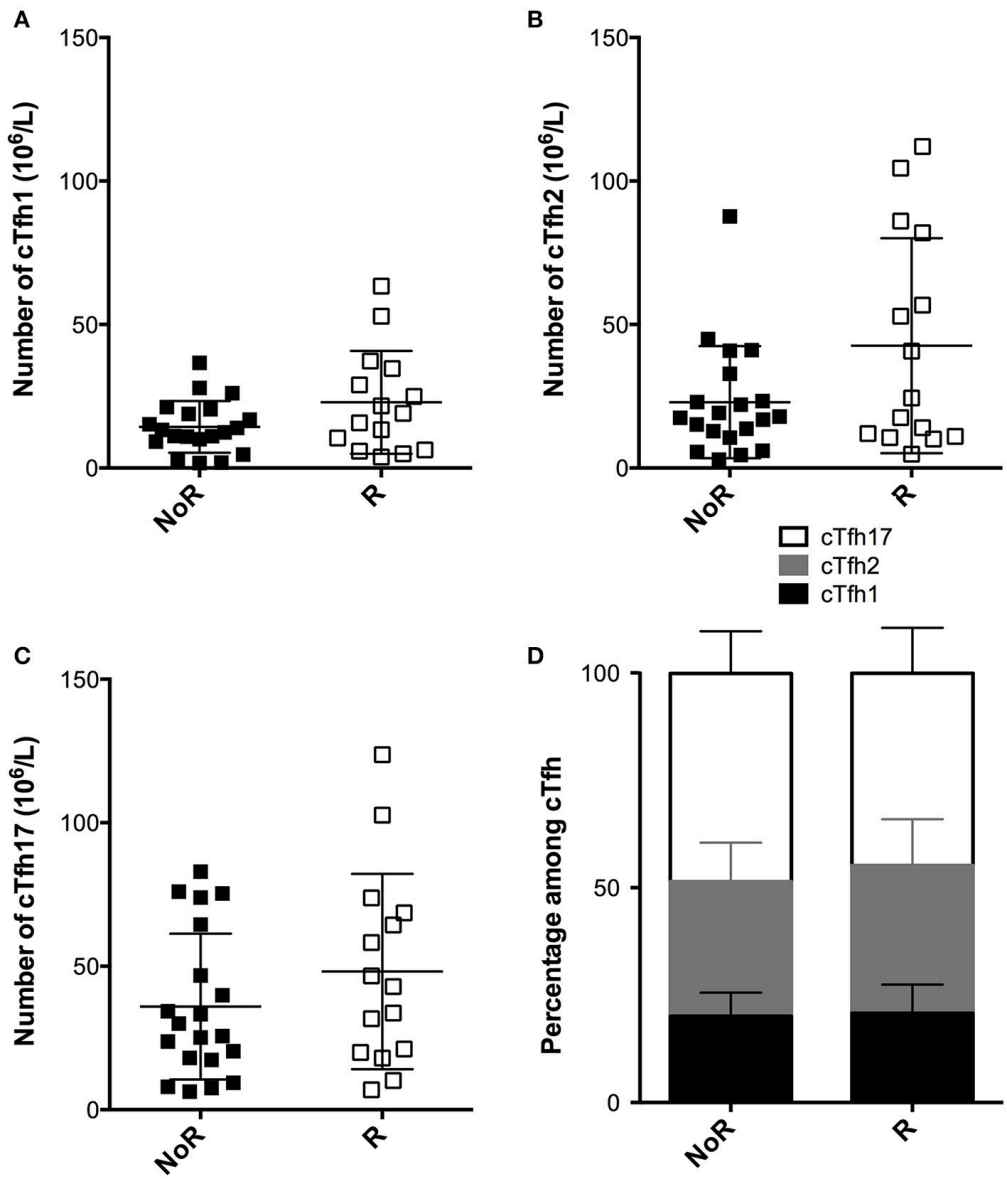
Comparison of the static profile of cTfh of responders and non-responders to A/Switzerland hemagglutinin. **(A–C)** PBMC of transplant patients were collected just before vaccination. The number cTfh of each of the three subsets (**A**: cTfh1; **B**: Tfh2; **C**: cTfh17) was enumerated by flow cytometry. Data from transplant patients that had developed (Responders, R; *n* = 15; open square) or not developed (non-responders, NoR; *n* = 20; black square) antibodies against Switzerland hemagglutinin are compared. Each symbol represents a patient, mean ± SD is indicated. **(D)** The proportion (mean ± SD) of each of the three subsets of cTfh is compared between the two groups. Mann-Whitney test.

Static analysis of the cTfh profile just prior vaccination showed poor performance for the discrimination of responders and non-responders. Indeed, neither the absolute number (Figures 6A–C), nor the proportion of the three cTfh subsets among CD4+T cells (Figure 6D) were statistically different between the two groups.

**Figure 7:**
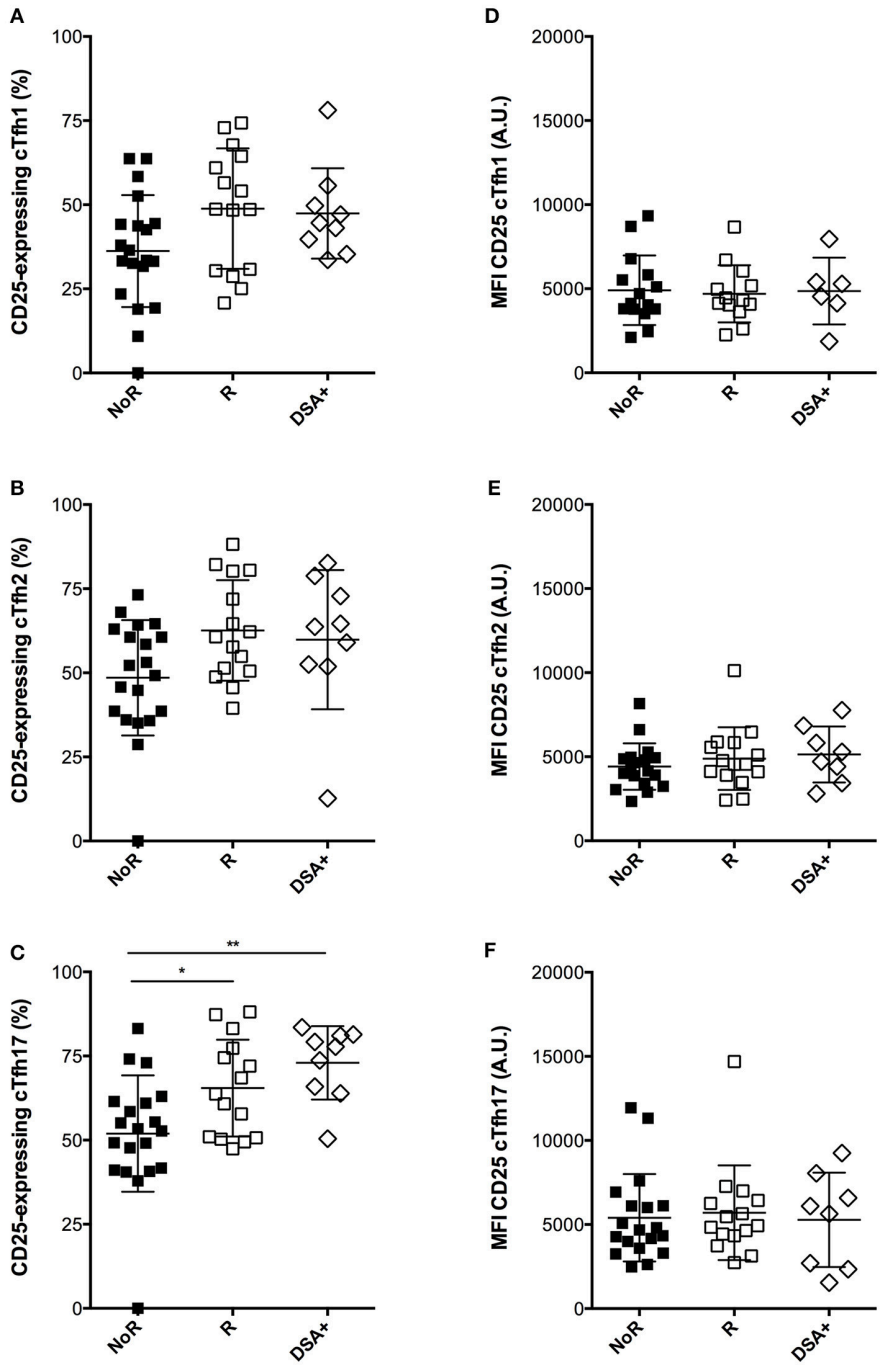
Comparison of the dynamic profile of cTfh of responders and non-responders to A/Switzerland hemagglutinin. **(A–C)** PBMC of transplant patients were collected just before vaccination. The percentage of CD25-expressing cTfh of each of the three subsets (**A**: cTfh1; **B**: Tfh2; **C**: cTfh17) was enumerated by flow cytometry after 24 h of *in vitro* stimulation. Data from transplant patients that had developed (Responders, R; *n* = 15; open square) or not developed (non-responders, NoR; *n* = 20; black square) antibodies against Switzerland hemagglutinin are compared between themselves and with that of a cohort of nine transplant recipients that had just developed DSA (group DSA+). **(D–F)** The level of expression of CD25 (as reflected by mean fluorescence intensity, MFI) was measured by flow cytometry after 24 h of *in vitro* stimulation for each of the three subsets of cTfh (**D**: cTfh1; **E**: Tfh2; **F**: cTfh17) and compared between the three groups. Each symbol represents a patient, mean ± SD is indicated. ^*^p < 0.05; ^**^p < 0.01; ANOVA, Dunn's multiple comparisons test.

In contrast, responders displayed a consistent trend for higher proportions of activated cTfh after overnight *in vitro* stimulation in presence of immunosuppressive drugs as compared with non-responders (Figures 7A–C). Importantly, this “residual activatability” reached statistical significance for cTfh17 (Figure 7C), the dominant subset in transplanted patients (Figure 1E) and the most efficient for TD antibody response (28). Of note, the intensity of CD25 expression by *in vitro* activated cTfh was not different between responders and non-responders (Figures 7D-F).

**Figure 8:**
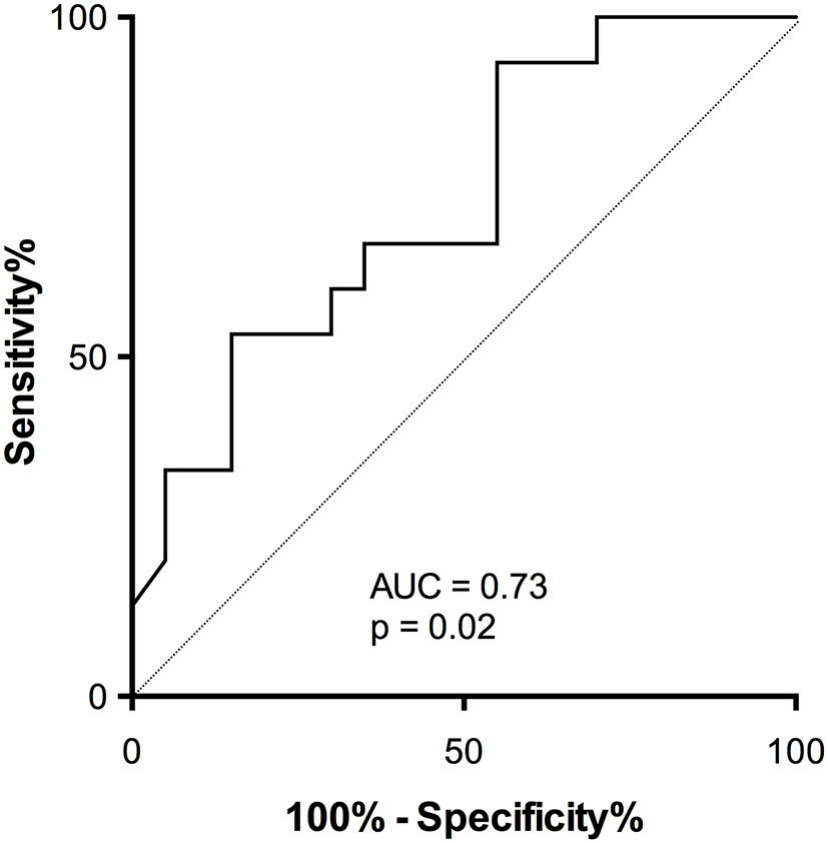
Accuracy of activatability of cTfh17 to predict antibody response in patients on therapeutic immunosuppression. The accuracy of residual activatability of cTfh17 to identify transplant patients able to mount a TD antibody response despite immunosuppressive therapy was appreciated by plotting the Receiver Operating Characteristic (ROC) curve.

ROC analysis was used to estimate the performance of the assay to discriminate responders from non-responders (Figure 8; AUC = 0.73, *p* = 0.02).

Finally, we retrospectively screened the Lyon University Hospital biobank and identified 9 renal transplant patients, for whom PBMC had been frozen at the time of *de novo* DSA appearance (clinical characteristics detailed in Table 3). Interestingly, these nine patients displayed the same profile of residual cTfh17 activatability as responders to influenza vaccine, a level that was significantly higher than non-responders to the vaccine (Figure 7, group DSA+).

**Table 3:** Characteristics of DSA+ patients.

	**DSA+ patients** ***n* = 9**
**CHARACTERISTICS AT TIME OF TRANSPLANTATION**	
**Recipient**	
Age at transplantation (years)	50.9 ± 19.5
Origin of renal disease, *n* (%)
Glomerulonephritis	2 (22%)
Diabetes mellitus	3 (33%)
Vascular	1 (11%)
Other	3 (33%)
Duration of dialysis (months)	18.7 ± 15.2
**DONOR**	
Donor age (years)	48.4 ± 25.2
Donor sex, men, *n* (%)	5 (56%)
Deceased donor, *n* (%)	9 (100%)
Extended criteria donor, *n* (%)	4 (44%)
**TRANSPLANTATION**	
Number of HLA-A, B, C, DR, DQ mismatches	7.3 ± 2.0
Combined kidney-pancreas transplantation, *n* (%)	2 (22%)
Cold ischemia time (min)	956 ± 320
Delayed graft function, *n* (%)	0
**IMMUNOSUPPRESSION**	
**Induction therapy:**	
Thymoglobuline, *n* (%)	8 (89%)
**Maintenance regimen:**	
Tacrolimus, *n* (%)	4 (44%)
Ciclosporin, *n* (%)	5 (56%)
mTOR-Inhibitors, *n* (%)	1 (11%)
Mycophenolate, *n* (%)	8 (89%)
Azathioprin, *n* (%)	1 (11%)
Prednisone, *n* (%)	9 (100%)
**CHARACTERISTICS AT TIME OF DSA DETECTION**	
Time elapsed since transplantation (months)	37.6 ± 57.9
eGFR (ml/min/m^2^)	54.1 ± 24.6
Proteinuria >0.5 g/24 h, *n* (%)	1 (11%)
Nb of DSA specificities	1.3 ± 0.5
Anti-HLA I/II/I+II	1 (11)/8 (89)/0
MFI of immunodominant DSA	4386 ± 6342
Time elapsed between DSA detection and cTfh profiling (months)	1.9 ± 2.0

*mTOR, mamalian target of rapamycin; eGFR, estimated glomerular filtration rate*.

## Discussion

In this translational study, we compared the characteristics of circulating Tfh (cTfh) of 36 renal recipients and nine healthy volunteers. We showed that while therapeutic immunosuppression tended to reduce the number of cTfh1 and 2 it had little impact on the number of cTf17, which was the dominant subset in renal recipients, regardless of the time elapsed since transplantation. Interestingly, previous studies have shown that Tfh17 are particularly potent inducers of antibody responses (28). The reason why Th17 subset seems less impacted by thymoglobulin-induced depletion than other T cell subsets is not clear but has been reported by others (35). Possible mechanisms include faster homeostatic proliferation due to the peculiarities of Th17 cell metabolism (36) and/or intrinsic resistance to complement-mediated lysis. Besides thymoglobulin, high-dose glucocorticoid can also induce T cells apoptosis through direct and indirect (cytokine suppression) effects. Interestingly, the different T helper subsets have distinct sensitivity to glucocorticoid: Th1 cells are susceptible to both direct steroid-induced apoptosis and cytokine suppression, Th2 are sensitive only to cytokine suppression, and Th17 are resistant to both steroid-mediated activities (37). Part of this resistance could be explained by the fact that Th17 cells stably express P-glycoprotein (P-gp)/multi-drug resistance type 1 (MDR1), which limits the number of steroid molecules that can enter these cells (38).

Therapeutic immunosuppression significantly reduced cTfh activation. As recently reported, tacrolimus appeared more efficient than ciclosporin (39). However, significant inter-individual variations were observed. Thus, the presence of immunosuppressive drugs was not able to block cTfh activation *in vitro* in ~20% renal transplant recipients, who exhibited the same percentage of CD25-positive Tfh as healthy volunteers (a parameter that we named “residual activatability”).

This led us to hypothesize that cTfh profiling might be used to identify renal transplant patients at risk for *de novo* DSA generation, an hypothesis supported by recently published works (22, 40). In the absence of efficient curative treatment of AMR (10, 12), such an assay would be of major clinical interest. It would allow optimization of primary prevention of DSA generation by guiding personalized adaptation of the immunosuppressive regimen before appearance of alloantibodies. In line with this concept is the fact that operationally tolerant patients, i.e., transplant patients maintaining stable renal graft function despite the fact they don't take immunosuppressive drugs, have been shown to have anergic cTfh (41). High-risk patients could for instance benefit from the introduction of belatacept, a new drug that has shown good efficacy in controlling B/Tfh crosstalk (42, 43).

*De novo* DSA generation is not only a relatively rare event (16, 17), it is also influenced by several factors [including graft immunogenicity: i.e., the number of epitope mismatches between donor and recipients, age of recipient…etc (15)], which creates high variability between donor and recipient pairs. This made the direct analysis of the correlation between cTfh profile and DSA appearance impossible in our relatively limited cohort. To circumvent this problem, we took advantage of the annual vaccination campaign against influenza virus, which offered several important advantages: (i) vaccine formulation consisted in a combination of viral protein, which are prototypical thymodependent antigens, (ii) all patients received the same antigenic challenge, (iii) the timing of immunization was known. Because our interest was in *de novo* generation (primary response) of DSA the analysis focussed on the response to hemagglutinin of the A/Switzerland strain, which was present in the vaccine formulation for the first time in 2015. Responders and non-responders did not differ regarding the main clinical characteristics, including immunosuppressive regimen. While the two groups could not be discriminated on the basis of the number of cTfh or their polarization profile, responders exhibited higher residual activatability for cTfh17 (i.e., higher upregulation of the activation marker CD25 on cTfh17 after *in vitro* activation). Interestingly, over the 3 years follow-up period that elapsed since the end of the study, two (5.5%) patients from the cohort developed *de novo* DSA and both were from the responder group. Furthermore, a pilot analysis conducted retrospectively on the PBMC of nine renal transplant patients that had developed *de novo* DSA, found similar residual activatability of cTfh17 in these patients.

In conclusion our study suggests that “residual activatability” of cTfh17 could be used to monitor non-invasively renal transplant patients under therapeutic immunosuppression and identify those at high risk for *de novo* DSA generation. These results pave the way for future large prospective studies that will assess the performance of this assay and its potential to guide personalized adaptation of immunosuppressive regimen and optimize prevention of DSA generation.

## Ethics Statement

The study was carried out in accordance with French legislation on biomedical research and the Declaration of Helsinki. The study was approved by the Comité de Protection des Personnes Sud-Est IV (ref#L15-166) and all patients signed a consent form to participate in this study.

## Author Contributions

Conception and design of the experiments: SD, CS, and OT. Acquisition, analysis, and Interpretation of data: SD, CS, MV, EB, NP, and OT. Drafting the manuscript: SD, CS, MV, EB, NP, BL, AK, GM, TD, EM, and OT.

### Conflict of Interest Statement

The authors declare that the research was conducted in the absence of any commercial or financial relationships that could be construed as a potential conflict of interest.
